# A regional One Health approach to mapping antimicrobial resistance interactions via systems thinking

**DOI:** 10.1186/s42522-025-00173-5

**Published:** 2025-10-06

**Authors:** Claudia Huebner, Nils-Olaf Huebner, Tillmann Goerig, Steffen Flessa

**Affiliations:** 1 Chair of General Business Administration and Health Care Management, University of Greifswald, F.-Loefflerstr. 70, 17489 Greifswald, Germany; 2 Institute of Hygiene and Environmental Health, University Medicine of Greifswald, W. - Rathenaustr. 49, 17489 Greifswald, Germany

**Keywords:** Antibiotic resistance, One Health region, Causal loop diagram, Systems thinking

## Abstract

**Background:**

Antimicrobial resistance (AMR), as an original One Health problem, combines inextricable interactions between the human, animal and environmental dimensions. Addressing this challenge requires systemic thinking and coordinated networking between different levels of society and regional institutions. Knowledge of causal relationships, their mutual influence and the ability to assess the impact of possible interventions are prerequisites for coherent action to combat the further spread of antimicrobial resistance in a region. An integrated regional approach has not yet been addressed in One Health research on antimicrobial resistance.

**Methods:**

This study is based on a systems thinking approach and uses a causal loop diagram to visualise the relationships between human, animal and ecological components in a circular AMR system map for a One Health model region. The participatory approach actively involved regional stakeholders in the data collection and modelling process through surveys, semi structured interviews and interactive workshops. Based on the developed causal loop diagram, leverage point analysis is applied to estimate which types of interventions would have the greatest ability to address antimicrobial resistance in the One Health region.

**Results:**

Our results show that the system mapping tool is suitable for demonstrating the relationships regarding AMR in the One Health context for a defined region. It provides an opportunity to identify and visualise important risk factors that are direct or indirect drivers of AMR. Specifically, two amplifying and two balancing loops have been constructed in the model, covering antibiotic stewardship, public awareness, regional data management and environmental impact. Interdisciplinary and intersectoral collaboration, homogeneity of data and public awareness were identified as important leverage points. The graphical illustration of the causal loop diagram enables political and economic decision-makers to develop a deeper understanding of regional resistance patterns and the rational use of antibiotics from a One Health perspective.

**Conclusion:**

This study is one of the first applications of a participatory systems thinking approach to the topic of AMR in the context of a One Health region.

**Trail registration:**

Not applicable.

## Background

Antimicrobial resistance (AMR) is no longer limited to the use of antibiotics in human and veterinary medicine. Rather, it combines inseparable interactions between the dimensions of humans, animals and the environment [1]. Although the environment has long been considered a neglected aspect of AMR, its influence is now recognized as an important reservoir for naturally occurring of antimicrobial resistance and for its spread through intensive misuse of ecological resources [2]. Of particular importance are inadequately treated waste and wastewater, livestock farming, agriculture and aquaculture, and environmental factors such as wildlife migration and the use of agrochemicals and heavy metals in soils and surface waters [3]. Accordingly, AMR is rightly defined as an original One Health problem that is recognized and addressed by the WHO [4, 5]. The One Health concept takes into account the interdependence of human, animal and environmental health and provides a comprehensive framework for understanding and addressing the complexity of AMR [6]. 

The interrelationships outlined above show that this complex and highly dynamic problem can no longer be solved with simple, locally applicable measures, but requires systemic thinking and coordinated cooperation between different levels of society and regional units [7]. It has long been proven that regions enable us to implement the concept of network governance [8]. Regionality and networking are essential components of an integrated approach, meaning that regional players should be consistently involved in all analyses and decisions. This applies not only to the healthcare system, science, politics, administration, and the economy but also to the population, from schoolchildren to pensioners. However, the literature to date shows clear deficits in the cooperation structures of One Health networks in terms of the diversity of stakeholder groups and sectors represented [9]. 

A systems-oriented approach enables the linking of different data sources, such as quantitative data sets, expert knowledge and stakeholder opinions from surveys or interviews. It follows a conceptual path of interactions between dynamic subsystems, which makes it possible to understand causal chains and identify mutual synergies and feedback loops. This allows conclusions to be drawn about how changes in individual variables and interventions affect subareas and the overall system [10]. Methods and tools for complex systems thinking are already widely used in public health research [11–13]. The most common objective hereby is to make the problem more tangible for political decision-makers through visualization. Qualitative system mapping and causal loop diagrams, in particular, have therefore become established as suitable methods for this purpose [14]. Causal loop diagrams describe feedback mechanisms that promote synergies that strengthen the system and simultaneously enable the identification of leverage points to interrupt or slow harmful feedback loops [15]. The analysis of leverage points is a useful method for assessing potentially effective interventions aimed at system change and sustainability transitions. An application of the tool to the topic of One Health research, particularly AMR, is quite possible, as a few publications have already shown [16, 17]. In particular, Matthiessen et al. were able to show in their work that the tool of system mapping, by connecting animals, humans and the environment in a model, makes it possible to address the issue of AMR holistically and contribute to the application of the ‘One Health’ concept [18]. 

The aim of this study was to develop a regional One Health approach to address the problem of AMR via systems thinking. The approach consists of identifying the key feedback loops between AMR and the environment, human and veterinary medicine, population participation and economy in a defined region and gaining insights into potential leverage points and interventions. A qualitative system mapping in the form of a causal loop diagram was used to illustrate these cause-effect relationships from the perspective of the One Health Region of Western Pomerania. The analysis includes a participatory approach as an established method to actively involve stakeholders in the data collection process [19]. 

## Methods

**Fig. 1:**
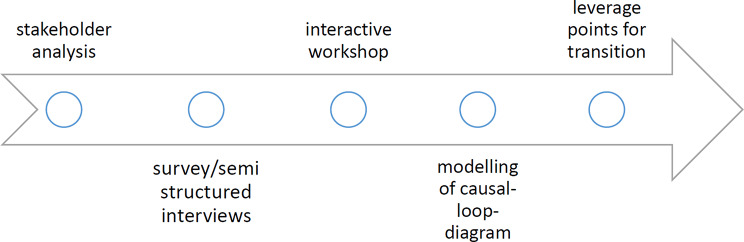
Study outline

The study was based on a participatory approach that actively involving regional stakeholders. The study outline is shown in Fig. 1. To include a range of perspectives on antibiotic resistance, an initial stakeholder analysis identified four main professional fields: healthcare providers, health insurance companies/medical associations, health authorities and research institutions. Additionally, organisations from agriculture and environmental protection sector were also addressed.

Modelling included primary data collected through surveys and supplementary semi-structured interviews. The surveys assessed the initial situation and consisted of questions about personal attitudes and levels of knowledge regarding One Health and AMR, potential collaboration in implementing strategies and suitable adoption factors. Selected aspects were then explored further in the interviews.

In preparation for the conceptualisation and development of the One-Health model, an interactive stakeholder workshop was held. It formed part of a hygiene event on the topic of ‘One Health’, organised by KOMPASS. The non-profit organisation KOMPASS is a regional MDRO-network that connects inpatient and outpatient healthcare facilities, public health services, medical laboratories and nursing services in the fight against the spread of MDROs. The network provides a forum for professional exchange, training and support in antibiotic and surveillance management. During the workshop, discussions took place in smaller, self-organised table groups. Topics for discussion were derived from the results of a previous survey. First, the workshop participants at each table recorded their thoughts in mind maps. This was followed by a moderated discussion between the groups. Contributions from the workshop participants were recorded jointly on flipcharts. The study group also took extensive notes, which formed the basis for subsequent data analysis process.

A targeted deductive thematic analysis was conducted for the data evaluation. This is a modification in the different steps to the method described by Braun and Clarke in 2006 [20]. First, we familiarised ourselves with the data by rereading the transcripts and linking them to the flipcharts and transcribed interviews. In the second step, we generated variables from the data. Each variable (equivalent to a category) refers to a relevant point from the AMR topic area. Thirdly, the subsystems (analogous to themes) were grouped based on the influencing variables assigned to them. When reviewing the clusters formed, variables necessary for the AMR-context we added, omitted, reworded or assigned to another subsystem. Next, we identified cause-and-effect chains to link the variables. Finally, the individual subsystems were combined into an overall cause diagram, focusing particularly on developing cross-connections between variables from different subsystems.

The regional One-Health concept is visualized in the causal loop diagram. In practice, the individual variables are connected by arrows that represent causal relationships. The arrows are given plus (+) or minus (-) polarities to indicate the nature of the relationship, i.e., whether they reinforce or diminish each other. Small parallel lines can be added to the arrows as lag markers to represent relationships with time lags. The variables are arranged in multiple loops, forming circles of cause and effect. The number of positive and negative polarities in a loop determins its behaviour. Positive loops (reinforcing loops, with only positive arrows or an even number of negative arrows) reinforce a process so that a change in any element continues to act in the same direction. They have the potential to create exponential growth in a system. In contrast, negative loops (balancing loops with odd numbers of negative arrows) counteract changes and tend to regulate growth. The causal loop diagram was created with Vensim^®^ (PLE version 10.2.2), a simulation software for the dynamic modelling of systems.

On the basis of Meadow’s theory [21], different levels of leverage effectiveness are categorised hierarchically. In this analysis, various leverage points are first listed and then evaluated and ranked in terms of their effectiveness.

## Results

### Participants

The survey resulted in 80 responses, 47 of which were from service providers (35 nurses and 12 doctors), 16 from health authorities, 14 from other areas of healthcare, 1 from health insurance companies and 2 from outside the healthcare sector. Fourteen experts participated in the interactive stakeholder workshop and five individual interviews were conducted and evaluated. The stakeholder groups to which the workshop and interview participants belong are shown in Table 1.

**Table 1:** Number of participants in the workshop and expert interviews broken down by stakeholder groups

Stakeholder group	Workshop participants	Inter viewees
Healthcare providers		
Hospital physician	2	
Practising doctor	1	
Laboratory	1	1
Pharmacy	1	
Health insurance	1	
Health authorities		
State Office for Health	3	
Association of Panel Dentists	1	1
Association of Statutory Health Insurance Physicians	1	1
State Office, Veterinary Medicines Monitoring Department		1
Research institutions		
Animal Health Research Institute	1	
University Medicine (One Health research group)	1	
Farmers’ Association	1	1
Total	14	5

### Survey and semi-structured interviews

The survey revealed that respondents already consider AMR to be of great importance in their everyday work. A very high proportion considered the proposal for regional and interprofessional cooperation to prevent the spread of MDROs to be effective. Similarly, implementing One Health structures to address health problems was considered effective. However, according to the interviewees, existing networks and working groups tend to work independently of each other and are primarily sector-specific.

Of the One Health topics, zoonoses and AMR in humans and animals were mentioned most frequently, followed by the impact of industry and agriculture, and environmental influences. In this context, existing prejudices and misrepresentations about the spread of AMR were highlighted, which the experts believe can only be eliminated through mutual exchange and education. A key issue raised by all experts is the exchange and mutual use of surveillance and antibiotic consumption data to inform cross-sectoral and cross-institutional prevention measures.

### Thematic analysis

The first three steps of the thematic analysis resulted in the identification of variables that could be grouped into four subsystems: antibiotic management, data management, environmental impact and public engagement (Table 2). Subsequently, variables were adjusted and linked according to the modelling process.

**Table 2:** Subsystems and associated model variables

Subsystem	Variables	Adjusted variables according to the modelling process
Antibiotic management	Antibiotic stewardship	antibiotic stewardship
	guidelines	guidelines
	prescription behaviour	prescription behaviour
	availability and reimbursement	antimicrobial resistance
	of antibiotics	surveillance*
data management	antibiotic stewardship/collaboration surveillance	surveillance* antibiotic stewardship
	quality of data (infrastructure, homogeneity) legal requirements (reporting obligations, data protection)	interdisciplinary and intersectoral collaboration* data infrastructure homogeneity of data*
environmental impact	livestock and arable farming soil and water contamination waste and wastewater treatment wildlife migration	livestock and arable farming* spread of liquid manure and dung soil and water contamination antimicrobial resistance
public engagement	awareness (campaigns, rethinking, education) resource responsibility willingness to pay consumer demand	campaigns, awareness resource responsibility rethinking* willingness to pay consumer demand offer of organic products

* variables evaluated as leverage points

### Causal loop diagram

**Fig. 2:**
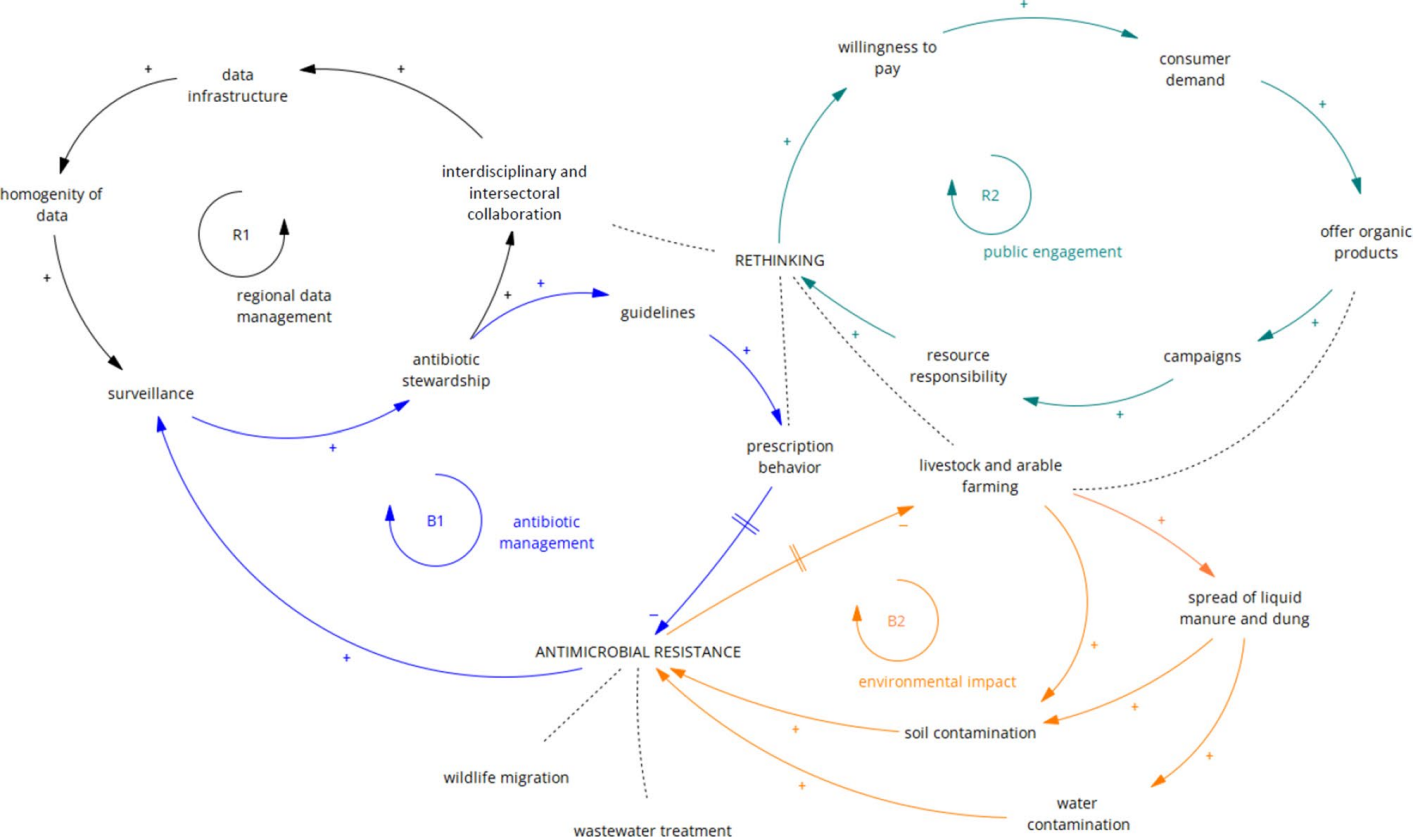
Causal loop diagram

The final causal loop diagram (Fig. 2) takes the four subsystems as loops, which are divided into two reinforcement loops (R) and two balancing loops (B).

The first loop (B1) is related to the use and handling of antibiotics in the clinical setting. Antibiotic consumption and resistance findings are recorded in surveillance databases, which are important tools for working in antibiotic stewardship programmes. Surveillance analyses and guidelines positively affect the antibiotic prescribing behaviour of doctors, dentists and veterinarians, including those in the outpatient sector. This, in turn, leads to an inhibitory, albeit delayed, effect on the spread of new resistance and is associated with the success of adequate antibiotic management.

A closely neighbouring, partially even overlapping subsystem is regional data management, which represents a reinforcing loop (R1). Again, well-documented surveillance provides a basis for successful antibiotic stewardship programmes and improves collaboration across disciplines and sectors. As part of this cooperation, it would be ideal if a common data infrastructure could be set up that would allow data to be easily shared and accessed. This will also promote data homogeneity and thus the pooling of different data sources for joint use and analysis.

The second balancing loop (B2) of our causal loop diagram focuses on the environmental impact of AMR, with particular reference to land use for livestock and agriculture, rather than other environmental influences such as wildlife transmission or climate change. Extensive livestock farming and the associated spread of liquid manure and dung in fields contaminate soil and water with MDROs. Another threat is the increased use of biocides, which enter the environment and thus contribute to the spread of AMR.

A last reinforcing loop (R2) focuses on the aspect of public awareness. To raise awareness of the One Health problem among the population, education is needed, for example, through campaigns and media appearances aimed at different target groups. This leads to greater responsibility for resources and, ideally, causes a rethinking and even a paradigm shift within the population. One result is a greater willingness to pay and thus increased private demand for sustainably produced food. As a result, companies have an incentive to switch to more organic products and offer them more often, which in turn receive a positive image thanks to appropriate campaigns. The loop R2 thus reinforces itself.

### Leverage points

**Fig. 3:**
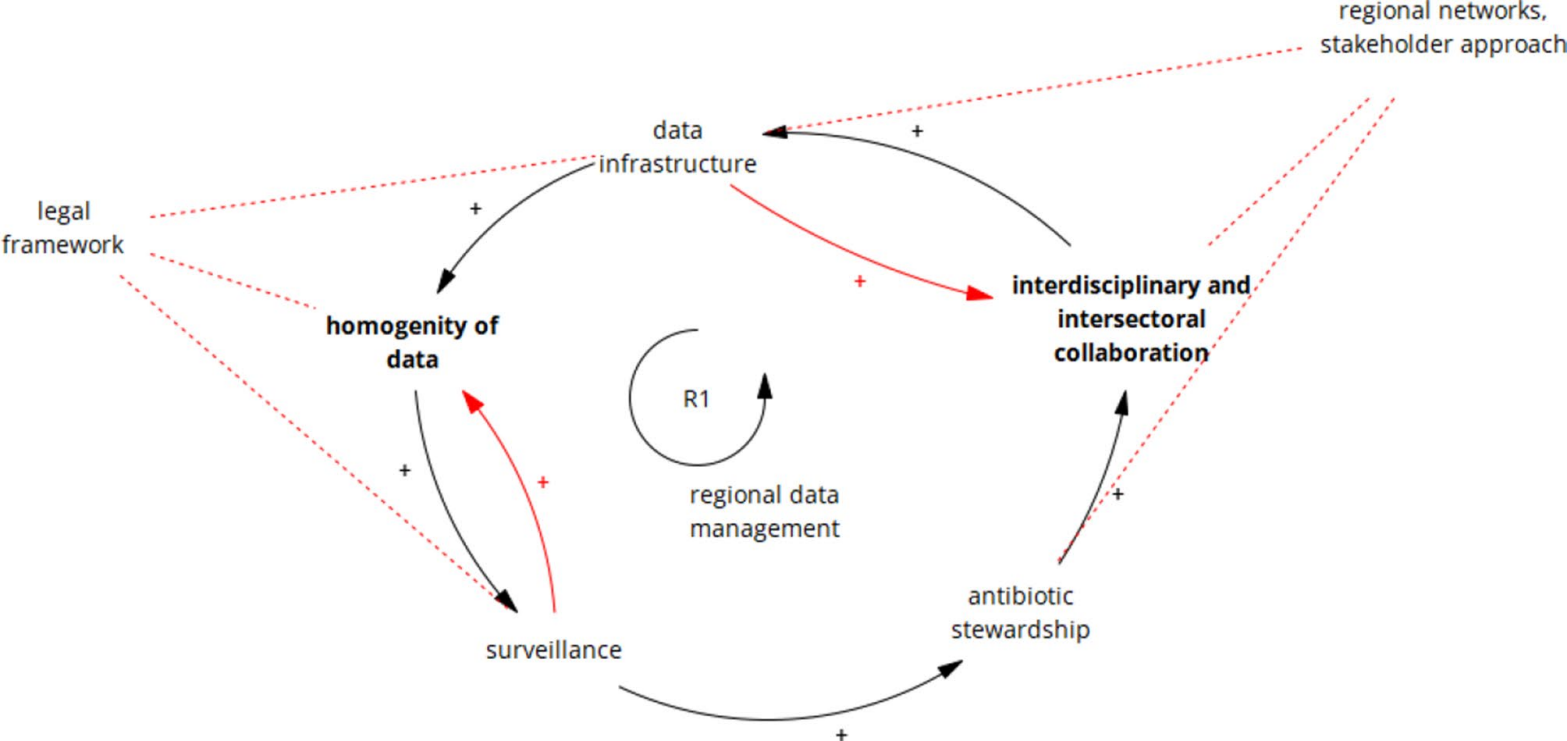
Extended scenario of leverage points in the subsystem “regional data management”

Several variables in the system model showed the potential to leverage system-wide change (see asterisks in Table 2). These leverage points vary in effectiveness according to the leverage point hierarchy. The two strongest leverage points, ‘interdisciplinary and intersectoral collaboration’ and ‘homogeneity of data’, were depicted in an extended scenario of the present causal loop diagram (Fig. 3). The development of regional networks in combination with the targeted involvement of stakeholders were identified as important interventions for successful collaboration. For better consolidation of existing data sources, more uniform specifications for data collection, documentation and storage through legal frameworks are necessary. In addition, bilateral feedback loops between the variables ‘data infrastructure’ and ‘collaboration’ as well as ‘surveillance’ and ‘data homogeneity’ were added to better highlight the interdependencies between each other.

A third strong starting point is ‘rethinking’ as a variable from the ‘public engagement’ subsystem. The aim is to increase people’s awareness to One Health, to encourage them to take responsibility and to empower them to take initiative. The necessary knowledge will be provided through targeted information campaigns and other educational programs.

## Discussion

The analysis translates knowledge about the multidimensional spread and containment of AMR from the perspective of a One Health region into an integrated systems approach. The model provides a comprehensive overview of several identified key factors related to regional antibiotic management and how these factors causally interact at different levels. In contrast to previous work on AMR in public health [16, 17], the environmental dimension has been given greater consideration. In particular, the links between livestock farming, aquaculture and agriculture and their impacts on the environment have been highlighted, as this is now considered one of the most important sources of resistance transmission in the environment [22]. Other environmental aspects, such as wildlife migration or waste and wastewater treatment, are only mentioned here as side effects. However, their importance in relation to antimicrobial resistance in the environment is being increasingly addressed in the literature [23, 24] and could be given greater emphasis in further model development.

The regional approach is central to our work. The systems model identified interdisciplinary and, in particular, intersectoral collaboration as a strong leverage point. Cooperation and regional networking are highly beneficial in the fight against the spread of AMR. This is already evident in the cooperation of MDRO-networks in different European countries led by the European Antimicrobial Resistance Surveillance Network (EARS-Net). Structures have been set up for network activities, particularly in the area of ​​surveillance, which could be used to expand the scope to include One Health content. Pantano and Friedrich from the regional MDRO-network Euregios have already briefly touched on this aspect, emphasizing the crucial role of the One Health concept in understanding the broader context of AMR [8]. Even more concrete is the RUmBA (Rational Use of Antibiotics in the Population) project in the One Health Region of West Pomerania [25]. Its aim is to transform the regional MDRO-network into a regional One Health network by recruiting additional stakeholders and integrating other, previously ignored One Health content into the network’s tasks.

A key prerequisite for successful cross-institutional and regional surveillance analysis is the merging of different data sources and settings. Integrating data from various sources within a single regional context could enhance efforts against AMR [26]. However, many of the institutions involved still need to overcome this major hurdle. The necessary homogeneity of the data can be guaranteed only once uniform standards for the collection, documentation and evaluation of data on antibiotic consumption and resistance have been established (lever point in loop R1). This requires appropriate government regulations (top-down approach), such as those already partially implemented in the reporting requirement. Empirical data from clinical and nonclinical settings form the basis of the surveillance analyses that underpin much of the work of antibiotic stewardship programs. A challenge is often the privacy policies of different organisational units, which often prevent the sharing of information between colleagues, especially in regard to building shared databases.

Another subsystem of our model approach is public engagement. Our surveys have shown that the problem of AMR is still greatly underestimated in the perception of the community. Efforts to date often focus only on the correct use of antibiotics by patients outside the clinical setting. The fact that AMR is a serious problem with social, environmental and economic consequences needs to be better understood and communicated. Information campaigns and targeted community involvement raise awareness and encourage changes in consumer behaviour, not only with regard to antibiotics but also with regard to other critical substances such as biocide-containing household cleaners or fertilizers for domestic use. Through appropriate educational campaigns, individuals can be empowered to strengthen their self-sufficiency (through a bottom-up approach). This has a strong influence on changing consumer behaviour through an increased sense of responsibility. This aspect has not yet been addressed in the models known to us. Grohn et al. mentioned only the factor “public awareness” in one loop but did not consider it further because it was outside the scope of the system model in their project [17]. Activating knowledge by involving the community in research helps increase public awareness and reduce the barrier between scientists and the public.

However, several limitations limit the validity of the modelling approach. First, the statements made by the stakeholders interviewed are very specific to their own work and are therefore difficult to generalize. This is particularly evident when the number of expert interviews conducted is small. However, a higher response rate could not be achieved due to the low level of feedback and the restriction to the region of West Pomerania. Second, the model represents only a selection of the most important statements. This means that individual points had to be omitted, which would have distorted the overall picture. Despite all the neutrality, the authors’ views are partly reflected in these choices. However, all decisions were discussed and made as a group in order to rule out individual opinions. In addition, other aspects have been deliberately included as side effects for modelling purposes but are likely to come to the fore later. These include the aspect of wastewater treatment and the influence of the spread of AMR through wildlife migration. Third, an attempt has been made to delineate certain areas to identify the loops. However, there is always overlap, so not all relationships can be shown.

The final causal diagram enables decision-makers to develop an in-depth understanding of regional resistance patterns and the rational use of antibiotics from a One Health perspective. Based on the leverage points identified in the model, initial policy recommendations for potential measures have been presented. However, further in-depth systems thinking analyses are required in order to map long-term effects in a multidimensional way.

## Conclusions

This study is one of the first to apply a participatory systems approach to the topic of AMR within a One Health region. It confirms that stakeholders should be involved in the targeted networking of One Health actors and organisations as early as possible, as this is an important issue for strategic health management. This will also help society develop a common understanding of the severity of the problem. Compared to previous models, a much more far-reaching analysis was carried out using systems thinking, so that the One Health dimensions of humans, animals and the environment were given equal consideration. The regional context was particularly important, as local characteristics in terms of resistance, data processing and social values were taken into account. The identified leverage points have a direct influence on this. However, the extent to which the model can be transferred or expanded to the national or international level is a matter of speculation and requires further modelling.

## Data Availability

No datasets were generated or analysed during the current study.

## Abbreviations

AMR: Atimicrobial resistance
MDRO: Multidrug-resistant organism
